# SEOM-GEMCAD-TTD clinical guidelines for localized rectal cancer (2021)

**DOI:** 10.1007/s12094-022-02816-9

**Published:** 2022-03-18

**Authors:** Jaume Capdevila, Ma Auxiliadora Gómez, Mónica Guillot, David Páez, Carles Pericay, Maria José Safont, Noelia Tarazona, Ruth Vera, Joana Vidal, Javier Sastre

**Affiliations:** 1 Department of Medical Oncology, Vall Hebron University Hospital, Vall Hebron Institute of Oncology (VHIO), Barcelona, Spain; 2 Department of Medical Oncology, Hospital Universitario Reina Sofía. IMIBIC. CIBERONC, Córdoba, Spain; 3 Department of Medical Oncology, Hospital Universitario Son Espases, Palma de Mallorca, Spain; 4 Department of Medical Oncology, Hospital de la Santa Creu i Sant Pau. U705. CIBERER, Barcelona, Spain; 5 Department of Medical Oncology, Hospital Universitari Mútua de Terrassa, Terrassa, Spain; 6 Department of Medical Oncology, Consorcio Hospital General Universitario de Valencia, Universidad de Valencia. CIBERONC, Valencia, Spain; 7 Department of Medical Oncology, INCLIVA Biomedical Research Institute, University of Valencia, Valencia, Spain; 8 Instituto de Salud Carlos III, CIBERONC, Madrid, Spain; 9 Department of Medical Oncology, Hospital Universitario de Navarra; Navarrabiomed, IDISNA, Pamplona, Spain; 10 Department of Medical Oncology, Hospital del Mar-IMIM, CIBERONC, Barcelona, Spain; 11 Department of Medical Oncology, Hospital Universitario Clínico San Carlos, Madrid, Spain

**Keywords:** Rectal cancer, Total neoadjuvant therapy, Guideline

## Abstract

The management of localized rectal cancer requires a multidisciplinary approach to optimize outcomes, reduce morbidity and prevent under or overtreatments. While early stages may obtain benefit of local resections without any additional therapies, locally advanced rectal cancer becomes a challenge defining the better sequential strategy of surgery, radiotherapy and chemotherapy. The latest results of international phase III studies have positioned the total neoadjuvant therapy as a potential new standard of care in high risk rectal cancers, however, the best schedule is still not well defined.

## Introduction

In Spain, colorectal cancer (CRC) is the most common type of cancer in both genders and the second cause of all cancer deaths. The incidence of CRC in 2021 has been estimated in 43,581 new cases. Of them, 14,209 have been cases of rectal cancer (8720 in men and 5489 women) [1]. Median age at the time of diagnosis is about 70 years. Global incidence of CRC is increasing mainly due to left-sided cancers in general and rectal cancer (RC) in particular.

Risk factors include age, diet (red or processed meat), alcohol and tobacco, overweight, physical inactivity, type II diabetes, inflammatory bowel disease (ulcerative colitis and Crohn`s) and family history of adenomas or CRC. The majority of cases of RC are sporadic. Hereditary component (lynch syndrome or familial adenomatous polyposis) is less frequent than in colon cancer.

The most common molecular pathway of RC development is chromosomal instability while approximately 13% are caused by a deficient mismatch repair.

The introduction screening programs (faecal occult blood test) have played roles to detect asymptomatic early stage and reducing mortality of CRC.

## Methodology

For developing this clinical guideline authors have reviewed and discussed most relevant literature published about RC. All the recommendations included in this guideline have had the consensus of all the authors and have been graded using “The Infectious Diseases Society of America-US Public Health Service Grading System” [2] (Table 1).

**Table 1:** Levels of evidence and grades of recommendation

Levels of evidence
I. Evidence from at least one large randomized, controlled trial of good methodological quality (low potential for bias) or meta-analyses of well-conducted randomized trials without heterogeneity
II. Small randomized trials or large randomized trials with a suspicion of bias (lower methodological quality) or meta-analyses of such trials or of trials with demonstrated heterogeneity
III. Prospective cohort studies
IV. Retrospective cohort studies or case–control studies
V. Studies without control group, case reports, expert opinions

Grades of recommendation
A. Strong evidence for efficacy with a substantial clinical benefit, strongly recommended
B. Strong or moderate evidence for efficacy but with a limited clinical benefit, generally recommended
C. Insufficient evidence for efficacy or benefit does not outweigh the risk or the disadvantages (adverse events, costs), optional
D. Moderate evidence against efficacy or for adverse outcome, generally not recommended
E. Strong evidence against efficacy or for adverse outcome, never recommended

## Diagnosis and staging

Most frequent warning signs for RC are rectal bleeding, tenesmus, and the change in bowel habit. 95% of cases are adenocarcinoma.

RC is defined as a tumour from the anal verge to 12–15 cm measured by rigid sigmoidoscopy or magnetic resonance imaging (MRI). According to the location is subdivided as low (up to 5 cm), middle (> 5 to 10 cm) and high (> 10 to 15 cm).

All patients must be discussed by a multidisciplinary team after diagnosis to individualize treatment.

After a suspicion, diagnostic procedures should include a complete anamnesis with family and personal history, physical examination including digital rectal examination, performance status, laboratory tests (complete blood count, liver and renal function, and serum level of carcinoembryonic antigen) [3].

Total colonoscopy/rectoscopy with biopsy is mandatory to confirm the diagnosis [3]. Virtual colonoscopy is an alternative if full colonoscopy is not feasible to rule out concomitant colon tumors; in case where complete colonoscopy cannot be carried out before surgery, it should be performed 3–6 months after surgery.

Thoraco-abdominopelvic computed tomography (CT) scan with intravenous contrast administration is the preferred study for evaluating the presence of distant metastases [3]. When a CT scan cannot be performed, chest X-ray and abdominal MRI should be considered.

Endorectal ultrasound (EUS) is recommended for evaluating tumour depth in early stages (cT1–T2) [4].

Rectal high-resolution MRI is the most accepted modality for preoperative local staging, determining the depth transmural tumour invasion, the status of the circumferential resection margin (CRM), the extramural venous invasion (EMVI), the height from the anorectal junction, the invasion other structures, the sphincter complex and the presence of suspicious regional nodes (has less sensitivity and specificity to evaluate lymph nodes).

MRI is recommended to plan surgical approach after neoadjuvant therapy in RC. CRM involvement is an independent prognostic factor for overall survival, local recurrence and disease-free survival. The presence of EMVI is associated with poor prognosis of local recurrence and disease-free survival.

Positron emission tomography (PET) is not recommended routinely for staging localized RC [4].

The 8th edition of the TNM staging system for rectal cancer should be used for clinical and histopathological staging [5]. T1 tumours have to be classified according to Haggitt and Kudo-Kikuchi stages depending on polyp morphology [6, 7].

After surgery, histopathological analysis of the sample should include: grade, quality of mesorectum, margins (proximal, distal and circumferential), depth of penetration (T), lymph, vascular and nerve invasion, number of regional lymph nodes (N), extranodal tumour deposits and response to neoadjuvant treatment.

Recommendations:

1. Multidisciplinary team is mandatory for individualized treatment (III, A).

2. Rectal MRI is the standard method for evaluating locally advanced rectal cancer. Endorectal ultrasound could be useful in early stage rectal cancers (III, A).

## Management of resectable localized disease

Radical resection of early stage RC (cT1/T2N0M0) should guarantee cure in these patients. Meta-analysis has shown that total mesorectal excision (TME) is equivalent to new local resection techniques (especially in cT1N0), but provides greater morbidity (sexual and urinary dysfunction) and the greater presence of a definitive stoma in distal tumors [8].

Studies and meta-analysis have placed transanal endoscopic microsurgery (TEM) as a technique of choice in cT1N0 rectal tumors [9], when they meet low-risk criteria[10] (low tumour grade, absence of lymphovascular and perineural invasion, and correct margin resection). Otherwise, TME should continue to be the treatment of choice for cT1, due to the high risk of local recurrence, although distant recurrence presents similar values between the two surgical techniques [11, 12].

Stage cT2N0M0 RC should be treated upfront with TME without perioperative treatments. However, if these tumors meet low-risk criteria, treatment with TEM associated with preoperative chemoradiotherapy (CRT) could be evaluated. If we treat these patients without neo(adjuvant) treatment, the risk of local recurrence is unacceptable, being around 30–50% [12].

The current evidence supporting TEM with perioperative CRT in cT2N0 RC comes from single series, prospective single arm phase II studies and meta-analyses, suggesting an acceptable local control (local recurrence (LR) between 4 and 7%) and no differences in distant metastases compared with TME [13–15]. Randomized clinical trials are currently ongoing to assess this strategy in early stage RC [16].

## Preoperative management of intermediate risk rectal cancer

TME surgery should be also proposed for those patients diagnosed with cT3 tumors without clear involvement of mesorectal fascia due to the high risk of recurrence and the high risk of mesorectal lymph node involvement [17].

Overall, intermediate risk patients (cT3 with very low, levators clear, MRF clear or cT1-3 in mid or high rectum, cN1 (not extranodal), no EMVI) benefit from preoperative treatment including either short-course radiotherapy (SCRT) or CRT based on fluoropyrimidines followed by high-quality TME. CRT usually consists of 28–30 fractions of 1.8 Gy with concurrent with fluorouracil-based chemotherapy followed by surgery after an interval of ≥ 6 weeks, while SCRT implies the delivery of 5 fractions of 5 Gy followed by surgery either 1 or up to 8 weeks later [18–20]. Both therapeutic approaches are equivalent in terms of survival, toxicity and clinical outcomes [21]. Therefore, CRT and SCRT are considered interchangeable, with a preference for the former when substantial tumour downsizing is needed to achieve clear resection margins or allow sphincter-sparing surgery [22, 23]. Adding oxaliplatin or targeted drugs to fluoropyrimidines in the neoadjuvant setting does not improve clinical outcomes and thus are not recommended [24–26].

### Preoperative management of high-risk rectal cancer

High-risk of recurrence rate RC defined by MRI includes the presence of extramural vascular invasion (EMVI), circumferential resection margin (CRM) threat or involvement, existence of enlarged lateral lymph nodes, tumour location in the lower third of the rectum and the high-risk TNM classification factors.

The subdivision of category T3 rectal cancer into two subgroups of extramural spread ≤ 5 mm or more than 5 mm resulted in significant different survival and local recurrence rates [27]. In a Norwegian study, the estimated rates of LR increased dramatically twofold with N stage from N0 to N2 and there was a fourfold increase in the rates of metastases from N0 to N2 [28].

The presence of EMVI is associated with poorer 3 years relapse-free survival (RFS) and a 3.7 times higher relative risk of developing metachronous metastases within 1 year of diagnosis [29, 30]. CRM is a powerful predictor of development of local recurrence, distant metastases and survival [31]. In the context of lateral nodal spread, LR rates can be as high as 35% [32]. Low tumour height is related to pelvic recurrence, worse 5-year overall survival (OS) and disease-free survival (DFS) rates [33, 34].

These risk factors, as well as obesity or gender, which may influence the quality of surgery, should be taken into account when deciding on preoperative treatment.

### Treatment selection

#### Preoperative chemoradiotherapy or short-course radiotherapy

A number of randomized trials have evaluated the effectiveness of the addition of chemotherapy to preoperative radiotherapy [35, 36]. Putative benefits of this therapeutic strategy include local radio-sensitization which may in turn reduce tumour volume, increase rates of pCR and facilitate a sphincter-sparing procedure.

Results from the German Rectal Cancer Study Group (the CAO/ARO/AIO-94 trial) demonstrated a benefit of preoperative CRT compared with postoperative treatment. Preoperative therapy was initially associated with significant improvements in LR (6% vs 13%; *p* = 0.006), sphincter-sparing surgery (39% vs 19%; *p* = 0.004) and treatment related toxicity (27% vs 40%; *p* = 0.001) [37]. Long-term follow-up confirmed the improvement in the 10-year cumulative LR (7.1% vs 10.1%; *p* = 0.048), although OS, DFS and the occurrence of distant metastases was similar in the two groups [19]. The EORTC2291 and FFCD9203 trials corroborated the local control benefit of preoperative CRT versus long-course RT alone but with no differences in survival [38, 39].

Preoperative CRT was then compared to SCRT in two randomized trials. In the Polish trial, CRT compared to SCRT demonstrated a higher pCR rate (16.1% vs 0.7%) and a lower positive circumferential resection margin rate (12.9% vs 4.4%)[40]. Similarly, a higher pCR was achieved with CRT (15% vs 1%) in the Trans-Tasman trial[21]. However, in both trials, there were no differences in LR rates or survival outcomes between the two preoperative treatments.

With respect to the type of CT administered concurrently with RT, a phase III randomized trial compared capecitabine (CPC)- or 5-fluorouracil (5FU) -based CRT either pre- or postoperatively demonstrated that CPC was non-inferior to continuous infusion of 5FU with regard to 5-year OS (75.7% vs 66.6%; *p* = 0.0004) [41].

Likewise, preoperative CPC CRT achieved similar rates of pCR, sphincter-sparing surgery, and surgical downstaging compared with continuous infusion of 5FU in the NSABP R-04 trial, which in a 2 × 2 design included 1608 patients with stage II or III RC. In this trial, the addition of oxaliplatin did not improve locoregional events, pCR, DFS, OS or surgical outcomes while toxicity was increased significantly [42, 43].

Similar results were seen when the addition of oxaliplatin to 5FU/RT or CPC/RT was evaluated in the STAR-01[24] and ACCORD 12/0405-Prodige 2[44] trials respectively. In the most recent trial published addressing this question, the PETACC 6 trial, preoperative oxaliplatin plus CPC-based CRT again impairs tolerability and does not improve efficacy [45].

In contrast, higher rates of pCR were seen in the oxaliplatin plus 5FU/RT arm in the CAO/ARO/AIO-04 trial (17% vs 13%; *p* = 0.038) [25]. The DFS at 3 years was 75.9% (95% CI, 72.4–79.5) in the oxaliplatin group versus 71.2% (95% CI 67·6–74·9) in the control group (hazard ratio [HR] 0.79, 95% CI 0.64–0.98; *p* = 0.03). Recently, results from the Chinese FOWARC phase III randomized trial found that oxaliplatin plus 5FU CRT although improved the pCR, no significant differences in 3-year DFS were detected [46].

Other randomized trials have also investigated the addition of targeted therapies to preoperative CRT for localized rectal cancer. However, further evidence is needed for a clear recommendation to add other agents to fluoropyrimidine-based CRT and how to integrate them within the total neoadjuvant therapy (TNT) approach.

The so called preoperative short-course radiotherapy (SCRT), 25 Gy over 5 days followed by immediate TME has demonstrated decreased rate of local recurrences compared to surgery alone[18]. No differences in rate of local recurrence or survival have been found when comparing both strategies, though a higher tumour downstaging were observed in favour of CRT [21, 40]. SCRT could be considered an alternative to CRT in intermediate-risk RC, and in high-risk RC for patients not suitable to receive a more intensive regimen due to comorbidity, age or poor performance status.

#### Total neoadjuvant therapy

Preoperative chemotherapy (CT) may be associated with better treatment compliance, may allow full systemic doses of CT to be delivered and an early micrometastases treatment. A recent meta-analysis shows that addition of preoperative CT to standard neoadjuvant chemoradiotherapy results in a higher pCR rate [47]. The optimal sequence of CRT/RT and CT is not well defined. The CAO/ARO/AIO-12 trial suggested a higher complete response rate after consolidation CT than after induction CT [48]. Data from OPRA trial report a higher organ preservation rate in the consolidation CT arm [49].

Three randomized trials have shown benefit of total neoadjuvant therapy (TNT) when comparing with standard treatment of CRT regarding 3-years DFS (induction mFOLFIRINOX followed of CRT vs CRT) [50], cumulative probability of disease-related treatment failure (SCRT followed of consolidation CAPOX/FOLFOX vs CRT) [51] and 3-years OS rate (SCRT followed of consolidation CAPOX/FOLFOX vs CRT) [52]. Inclusion criteria for these phase III trials were slightly different and increases the complexity of patient selection. The French study included stage II-III RC, the international RAPIDO trial recruited patients with high-risk RC defined y MRI bas cT4a or cT4b, EMVI, cN2, involved mesorectal fascia, or enlarged lateral lymph nodes, and finally the Polish study randomized patients with primary or locally recurrent RC involving adjacent organs (cT4) or a palpably fixed cT3.

On the basis of these results, in front of a high-risk RC, a TNT scheme might be considered in the setting of a multidisciplinary discussion and a case by case decision (Table 2).

**Table 2:** Main randomized trials of TNT

Study	N	Eligibility	Treatment strategy	Primary endpoint	Primary outcome	pCR	DFS
Marechal et al. [53]	57	cT2-T4/N +	mFOLFOX × 2 -CRT-TME vs. CRT-TME	ypT0-1 N0	32% vs. 34%*	25% vs. 28%*	NR
GCR 3 [54, 55]	108	≥ cT3, N + , EMVI + MRF + or distal	CAPOX × 4 -CRT-TME vs. CRT—TME—CAPOX × 4	pCR	14% vs. 13%*	NR	62% vs. 64%*
WAIT [56]	49	cT3-T4 or N +	CRT—5FU × 3 -TME vs. CRT—TME	pCR	16% vs. 25%*	NR	NR
KCSG CO 14–03 [57]	110	cT3-T4	CRT—CAPOX × 2—TME vs. CRT -TME	ypT0-2 N0	36% vs. 21%	14% vs. 6%*	NR
POLISH II [52, 58]	515	Fixed cT3 or cT4	SCRT- FOLFOX4 × 3 -TME vs. CRT (FOLFOX) -TME	R0 resection	77% vs. 71%*	16% vs. 12%*	43% vs. 41%*
KIR [59]	180	cT2/3 and N + EMVI + , or MRF + HDRBT	FOLFOX × 6—HDRBT vs. HDRBT	Chemo compliance	80% vs. 53%	31% vs28%*	72% vs. 68%*
STELLAR SPS:refid::bib60(60)	599	Distal or middle third T3-T4 and/or N +	SCRT—CAPOXX4—TME ± CAPOX X2 vs CRT -TME ± CAPOX X6	3-year DFS	64% vs. 62.3%	22.5% vs. 12.6%	64% vs. 62.3%
RAPIDO [51]	912	cT4a or cT4b, EMVI, cN2, MRF + or enlarged lateral lymph nodes	SCRT -CAPOX × 6 / FOLFOX4 × 9 -TME vs. LCRT -TME -CAPOX × 8 / FOLFOX4 × 12	3-year disease-related treatment failure	23.7% vs 30.4%	28% vs. 14%	23.7% vs 30.4%
PRODIGE 23 [50]	461	cT3-T4	mFOLFIRINOX × 12—CRT—TME -mFOLFOX × 6/Cape × 4 vs. CRT –TME—mFOLFOX6 × 12/Cape × 8	3-year DFS	76% vs. 69%	28% vs. 12%	76% vs. 69%

*DFS* disease-free survival, *pCR* pathological complete response, *CRT* chemoradiotherapy, *EMVI* extramural venous invasion, *MRF* mesorectal fascia, *HDRBT* high-dose rate endorectal brachytherapy, *NR* not reported

*Non-statistically significant

Nevertheless, although results of TNT seem favourable, some issues must be clarified in future trials:

- Chemotherapy regimen to add to neoadjuvant radiotherapy
- Type of radiotherapy: SCRT vs CRT
- Induction CT (before radiotherapy) vs consolidation CT (after radiotherapy)
- Subgroup of patients who benefit from TNT
- Selective radiotherapy
- Non-operative management

Recommendations:

1. Preoperative SCRT or continuous intravenous infusions of 5FU or oral capecitabine during CRT are recommended for patients with stage II or III rectal cancer [I, A]
2. The addition of oxaliplatin or to preoperative CRT is not recommended [I, D]

## Postoperative management of intermediate and high-risk rectal cancer

### Adjuvant chemotherapy

The evidence on the role of adjuvant CT in RC is limited. The vast majority of studies evaluating the use of adjuvant CT after preoperative CRT and total TME failed to demonstrate a benefit in PFS or OS, although they suffer from many challenges (old 5FU-based schedules, poor patient accrual and low compliance) [61–64]. A meta-analysis of these studies also failed to demonstrate a significant benefit [65]. Only 2 subsequent studies evaluating the addition of oxaliplatin to adjuvant fluoropyrimidine therapy have demonstrated a modest increase in PFS. The German phase III study CAO/ARO/AIO-04 examines the addition of oxaliplatin to both neoadjuvant and adjuvant therapy [25]. The long-term results of the randomized phase II ADORE study demonstrated a significant PFS benefit, although the OS benefit is limited to patients with ypN2 and minimally regressed tumours [66].

It is also unclear whether the benefit of adjuvant CT depends on the response to previous CRT. Postoperative pathological staging (ypTNM) may predict a high risk of subsequent local and distant recurrence, but there is no automatic benefit from the use of adjuvant CT. A pooled analysis of 3313 patients observed that those with a pCR after CRT may not benefit from adjuvant CT, whereas patients with residual tumour had superior outcomes when this treatment was administered although the test for interaction did not reach statistical significance [67].

### Adjuvant chemoradiotherapy

Neoadjuvant treatment (CRT or SCRT) has better outcomes than postoperative CRT with concomitant fluoropyrimidine-based chemotherapy after immediate radical TME, so adjuvant CRT is no longer recommended as a standard of care[37]. Only in those scenarios that are included in the below recommendations, adjuvant CRT may play a role.

Recommendations:

1. It is reasonable to consider adjuvant CT after preoperative CRT in patients with high-risk yp stage II and III (II, C). In the absence of more solid results, the decision to use adjuvant CT (fluoropyrimidines alone or in combination with oxaliplatin) should be evaluated considering the risk of relapse and potential toxicity. This option should be assessed on a personalized basis with each patient. For patients who are frail, with significant comorbidities, or with life expectancy of less than 5 years, CT should be omitted.

2. Adjuvant CRT could be used in patients with unexpected adverse histopathological features after primary surgery as positive CRM, pT4b, incomplete mesorectal resection, pN2 extracapsular spread close to MRF or extranodal deposits or in other cases with high-risk of LR if preoperative RT was not given.

## Non-operative management of localized rectal cancer

The pCR and cure rates have increased in the last years in patients with RC thanks to the improvement of neoadjuvant treatment strategies and TME. However, long-term functional sequelae including sexual and urinary dysfunction has been reported in more of half of patients with a permanent colostomy [68, 69]. In addition, 40% of patients with bowel continuity preservation describes a significant reduction in their quality of life (QoL) due to altered bowel function in the frequency, consistency, unpredictability or faecal incontinence [70–72]. For those patients achieving a complete clinical response (cCR) after a preoperative treatment, a close surveillance strategy (so called, non-operative management or watch-and-wait (W&W)) has been proposed as an alternative to rectal surgery with the benefits of a proctectomy sparing approach.

New international consensus criteria describe cCR as (a) the absence of any palpable tumour at digital rectal examination (DRE) and rectoscopy (except a small residual erythematous ulcer or scar) and (b) a substantial downsizing in the MRI with no observable residual tumour or residual fibrosis only and no suspicious lymph nodes. Endoscopic biopsy is only recommended when DRE and MRI are not conclusive [73].

Habr-Gama pioneered the W&W strategy more than 20 years ago in a prospective unicentric study for patients with a cCR after CRT with 5-FU, Leucovorin plus 50,4 Gy radiotherapy[74]. Authors demonstrated the safety of this approach with LR treated with savage surgery and no negative impact on RFS or OS. Currently, this treatment paradigm is of growing interest worldwide. The evidence from non-randomized studies in highly-specialized centers [75–77], as well as systematic reviews and meta-analysis [78, 79] show that avoiding surgery in patients with a cCR is safe keeping the surgery for those patients with tumour regrowth during follow-up. Recently, a large international multicenter registry (the International Watch & Wait Database) included more than 1000 patients with RC from 15 countries [80] that received neoadjuvant treatment and were managed by W&W strategy. At a median follow-up of 3.3 years, the 2-year cumulative incidence of local regrowth was 25.2% (95% CI 22.2–28.5%). Distant metastases were diagnosed in 8% of patients and 5-year OS was 85% (95% CI 80.9–87.7%). Discordantly, a retrospective case series analysis from Memorial Sloan Kettering Cancer center group showed that patients who avoid surgery after a cCR had worse survival and a higher incidence of distant metastases compared to patients who underwent to surgery after neoadjuvant treatment [81]. Again, this data comes from a retrospective non-randomized observational study including elderly patients and high prevalence of low rectal tumors. Currently, prospective clinical trials are ongoing to interrogate the organ preservation strategy according to clinical response after TNT [82].

Till now, no evidence from randomized trials is available to confirm both the long-term oncological outcomes and the superiority of organ preservation in terms of QoL. Currently, a recommendation for non-operative management after neoadjuvant treatment cannot formally be proposed and the W&W strategy may be reserved for prospective clinical trials and individually selected patients after a multidisciplinary evaluation of response.

## Management of unresectable rectal cancer

A RC involving adjacent non-resectable structures such as the proximal sacrum, pelvic sidewall, pelvic floor, prostate, or base of the urinary bladder or palpably fixed is considered not optimal for a complete and curative resection. One randomized trial comparing RT versus CRT showed favourable results in favour of CRT [83]. Data from Polish trial, which includes palpably fixed rectal tumours, showed a benefit in 3-years OS in patients treated with SCRT and consolidation oxaliplatin based CT [52].

## Local relapse

The incidence of LR has decreased significantly with the advances in multimodality treatment, but almost 4–10% of rectal cancer patients will develop LR disease which prognosis is poor with a median survival about 1–2 years. MRI is the optimal imaging modality for the assessment but there is no standard classification system of LR and treatment is very heterogeneous between centers [84]. Because up to 74% of patients will present LR with synchronous distant metastatic disease, all patient with suspected LR should undergo a full clinical staging evaluation. Treatment remains a major concern and it has to be discussed by a specialized multidisciplinary team taken into consideration prior therapy, the local extent of the recurrence, and whether distant metastases are present or not.

Recommendation:

- Surgery
  Surgery can be performed in a small number of cases (<20% in the best series). A complete resection (R0 with negative margins) is the most important prognostic factor and whenever possible, an attempt should be made to remove the tumour and affected organs [III, C]. When radical resection is achieved, 3-year DFS is approximately 57% and 3-year OS between 48 and 65% [85].
- Combined modality therapy

For most cases of LR, we suggest combined modality therapy rather than surgery alone. The specific approach depends on the previous treatment:

For previously irradiated patients, LR is habitually not easily resectable and reirradiation combined or not with chemotherapy could be an option. Re-irradiation is feasible in selected patients and may permit surgical salvage and long-time survival [IV, C]. New techniques as intensity-modulated RT (IMRT), proton beam irradiation or stereotactic body radiation therapy (SBRT) could be used in selected centres and have shown a low rate of acute toxicity (10–20%), good symptomatic relief (82–100%) and an acceptable incidence of late complications with median survivals about 40-60 months. When surgical option or re-irradiate is not possible, systemic palliative CT may be used to downstage the tumour, but efficacy is limited [V, C] [86, 87].

For previously unirradiated patients, management should be similar to that of newly diagnosed tumors, with neoadjuvant therapy prior to surgery [III, A]. In centres with experience, intraoperative radiotherapy could also be considered [88].

For patients not candidate for potentially curative multimodality therapy, symptom relief can often be achieved through a diverting colostomy, endoscopic laser ablation, stent placement or palliative radiotherapy [V, C].

### Follow up

About 25–40% of patients who present stage II or III of rectal cancer will develop recurrence. It is well reported than more than 90% of recurrences occur in the first 5 years after surgery and most of them within the first 3 years. Additionally, approximately 7% of patients will present with metachronous colon tumors.

Surveillance programs are generally based on physical examination, CEA evaluation, imaging and endoscopy, but the best follow-up strategy is not established (specific tests and inter-test interval). The most recent Cochrane analysis comparing *less versus intensive* follow-up, found that salvage surgery with curative intent was more frequent with intensive surveillance but this did not appear to translate into a survival advantage. Nevertheless, in line with most expert groups and published guidelines, we recommend intensive postoperative surveillance for most patients with resected stage II or III rectal cancer who would be considered candidates for curative-intent surgery. We also suggest not practicing any post-treatment surveillance for asymptomatic *stage I* rectal cancer except of interval colonoscopy. Besides, the surveillance strategy for resected *stage IV* disease should be individualized [22, 89].

Recommendation:

- Clinical assessment and CEA determination every 3–6 months for the first 2 years, and then every 6 months for a total of 5 years [V, D]
- Annual computed tomography (CT) of the chest, abdomen, and pelvis for 5 years [V, B]
- Colonoscopy is recommended at approximately 1 year following resection (or at approximately 3–6 months post-resection if not performed preoperatively due to an obstructing lesion). Repeat colonoscopy is typically recommended at 3 years, and then every 5 years thereafter, unless follow-up colonoscopy indicates advanced adenoma (villous polyp, polyp > 1 cm, or high-grade dysplasia), in which case colonoscopy should be repeated in 1 year [I, A].
- For patients with rectal cancer treated with transanal local excision only or those who have undergone low anterior resection and who have not received pelvic radiation therapy (RT), we suggest flexible proctosigmoidoscopy every 6 months for 3–5 years.

**Fig. 1:**
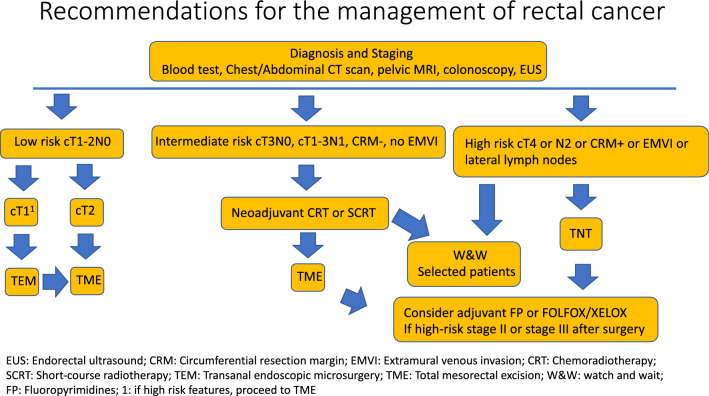
Recommendations for the management of rectal cancer

We do not recommend for routine surveillance: faecal occult blood testing, liver function tests, complete blood count, chest radiograph, positron emission tomography (PET) scans or ctDNA assays (Fig. 1).

Surveillance should be guided by presumed risk of recurrence and functional status of the patient. Patients at higher risk should be considered for more frequent testing. Additionally, if the patient is not a surgical candidate or a candidate for systemic therapy because of severe comorbid conditions or advanced age, surveillance tests should not be performed [90].
