# Correction to: SEOM‑GEMCAD‑TTD clinical guidelines for localized rectal cancer (2021)

**DOI:** 10.1007/s12094-022-02855-2

**Published:** 2022-05-20

**Authors:** Jaume Capdevila, Ma Auxiliadora Gómez, Mónica Guillot, David Páez, Carles Pericay, Maria José Safont, Noelia Tarazona, Ruth Vera, Joana Vidal, Javier Sastre

**Affiliations:** 1 Department of Medical Oncology, Vall Hebron University Hospital, Vall Hebron Institute of Oncology (VHIO), Barcelona, Spain; 2 Department of Medical Oncology, Hospital Universitario Reina Sofía. IMIBIC. CIBERONC, Córdoba, Spain; 3 Department of Medical Oncology, Hospital Universitario Son Espases, Palma de Mallorca, Spain; 4 Department of Medical Oncology, Hospital de la Santa Creu i Sant Pau. U705. CIBERER, Barcelona, Spain; 5 Department of Medical Oncology, Hospital Universitari Mútua de Terrassa, Terrassa, Spain; 6 Department of Medical Oncology, Consorcio Hospital General Universitario de Valencia, Universidad de Valencia. CIBERONC, Valencia, Spain; 7 Department of Medical Oncology, INCLIVA Biomedical Research Institute, University of Valencia, Valencia, Spain; 8 Instituto de Salud Carlos III, CIBERONC, Madrid, Spain; 9 Department of Medical Oncology, Hospital Universitario de Navarra; Navarrabiomed, IDISNA, Pamplona, Spain; 10 Department of Medical Oncology, Hospital del Mar-IMIM, CIBERONC, Barcelona, Spain; 11 Department of Medical Oncology, Hospital Universitario Clínico San Carlos, Madrid, Spain

## Correction to: Clinical and Translational Oncology (2022) 24:646–657 10.1007/s12094-022-02816-9

In Fig. 1 of this article an arrow was misplaced; the corrected Fig. 1 should have appeared as shown below.

**Fig. 1:**
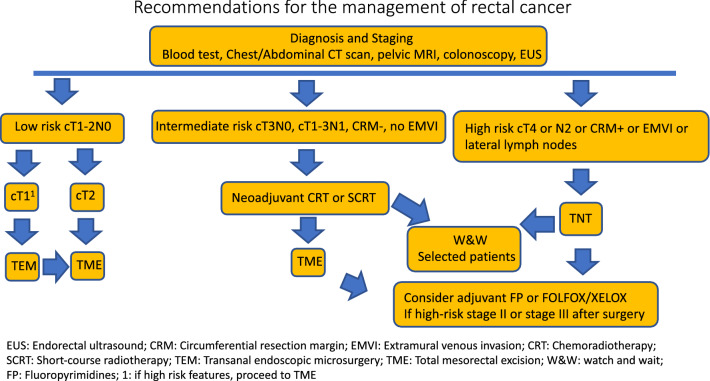
Recommendations for the management of rectal cancer

